# Revealing roles of competing local structural orderings in crystallization of polymorphic systems

**DOI:** 10.1126/sciadv.aaw8938

**Published:** 2020-07-01

**Authors:** Minhuan Li, Yanshuang Chen, Hajime Tanaka, Peng Tan

**Affiliations:** 1 State Key Laboratory of Surface Physics and Department of Physics, Fudan University, Shanghai 200433, China.; 2 Department of Fundamental Engineering, Institute of Industrial Science, University of Tokyo, 4-6-1 Komaba, Meguro-ku, Tokyo 153-8505, Japan.

## Abstract

Most systems have more than two stable crystalline states in the phase diagram, which is known as polymorphism. Crystallization in such a system is often under strong influence of competing orderings linked to those crystals. However, how such competition affects crystal nucleation and ordering toward the final crystalline state is largely unknown. This is primarily because the competition takes place locally and thus is masked by large positional fluctuations. We develop a unique method to correctly identify local symmetries by removing their distortions due to positional fluctuations. This allows us to experimentally access the spatiotemporal fluctuations of local symmetries at a single-particle level in crystallization of a charged colloidal system near the body-centered cubic–face-centered cubic border. Thus, we successfully reveal the crucial roles of competing ordering in the initial selection of polymorphs and the final grain boundary motion toward the most stable state from a microscopic perspective.

## Teaser

We reveal a spatiotemporal fluctuation of local orders inside intermediates during colloidal crystallization.

## INTRODUCTION

The crystallization kinetics is one of the most fundamental and important issues in condensed matter physics and materials sciences. Usually, the crystallization kinetics includes the generation of crystal nuclei from a supercooled liquid state and their further growth to large grains, forming a polycrystalline structure. This problem has attracted considerable attention both theoretically (*1*–*4*) and experimentally (*5*). Two-step crystallization driven by initial densification was proposed to play a crucial role in crystal nucleation (*6*–*10*). Contrary to this scenario, it has recently been shown that a supercooled liquid tends to form local structures with the same density as the liquid, yet whose rotational symmetry is broken to be consistent with those of crystals, and these structures act as precursors for future crystal nucleation (*11*–*17*).

In general, there are more than two stable crystals in the phase diagram, which is known as polymorphism. The famous examples include polymorphism of important materials such as carbon, water, and silica (*18*). In these systems, particularly, near the phase boundary between different crystals in the phase diagram, crystal nuclei are composed of competing multiple metastable solids (*19*), which should be considered as intermediate structures evolving toward the stable solid (*12*–*14*, *16*, *20*–*22*). Furthermore, the growth of crystal nuclei results in the formation of a polycrystalline mosaic structure, where crystalline grains are separated by grain boundaries (*23*–*30*), which can also be regarded as intermediate structures and whose slow migrations play a crucial role in the later coarsening and ripening process toward the most stable crystal polymorph (*31*–*35*). These complex intermediates, which are a transient state between a liquid and a solid state, play a critical role in the crystallization kinetics. They have local orientational order, but little translational order. This is a manifestation of the kinetic pathway of the ordering of a liquid to a crystal, which is preceded by local orientational ordering and then later followed by translational ordering (*36*). However, this scenario leaves us big puzzles about their precise roles: How is the formation of a stable solid realized by passing through such complex intermediates under competing ordering toward different crystals? Is there any universal nature of the intermediates that facilitate the transition? Thus, uncovering the nature of the intermediates and clarifying their roles in polymorph selection at a microscopic level are essential for answering these fundamental questions and reaching a full understanding of the crystallization kinetics under competing orderings.

Thanks to the appropriate particle size, time scale, and tunable potential, colloidal systems enable experimental observation of transition kinetics at a single-particle level (*5*, *14*, *17*, *37*–*43*). However, as shown in our previous works (*14*, *17*), many factors, such as thermal fluctuations and spatial mixing of different polymorphs, can cause serious distortions of the underlying local structural orders, preventing their proper identifications. Such distortion is a common source of difficulties in local structural identifications for any structure analysis methods (*44*), including wave-number space analysis (*45*), common neighbor analysis (*46*, *47*), Voronoi index analysis (*48*–*50*), and bond-orientational order approach (*51*, *52*).

Thus, a local structural analysis method resistive to such structural distortions due to positional fluctuations is important particularly in identification of structures that are under competing orderings and thus lack of well-developed translational order, such as precursor structures in a metastable supercooled liquid and structures in a grain-boundary region. To overcome this difficulty, we improve a structural analysis method based on bond orientation orders by removing its fragility against positional fluctuations of particles.

Here, we show how and where our new method could work and demonstrate its power to unveil intrinsic structural characteristics of intermediate states of crystallization, which are usually hidden behind thermal positional fluctuations. More specifically, by using a charged colloidal system with polymorphism as a model, we experimentally reveal that the intermediate structures are generally a mixture of local orders of different symmetries under an influence of strong positional fluctuations. Furthermore, we find that those local structures are actually short-lived and frequently transform among them. This spatial coexistence, together with newly found temporal fluctuations of competing orders, is a manifestation of the intrinsic spatiotemporally fluctuating nature of crystal precursors. This is natural, on noting that precursors are the intermediates between a disordered liquid and an ordered crystal and lack of translational order. Upon crystal nucleation, a specific type of order selected from the coexisting local orders in terms of stability starts to grow while increasing its lifetime, promoting the growth toward the corresponding crystal polymorph. The elucidation of this kinetic pathway allows us to understand the precise roles of the static and dynamical nature of precursors in crystal nucleation events in polymorphic systems. We also find that similar spatiotemporal fluctuations of local orders persist also in the grain boundary regions of the metastable solids, which makes the solid-solid interface rough and induce the boundary migration toward a more stable state. These results indicate that the microscopic process of ordering is a rather continuous stochastic process under thermal fluctuations from less stable to more stable states, accompanying the continuous increase in the spatial coherency and the lifetime of the most stable order. Our finding provides a simple general picture on the crucial role of intermediate states in determining the kinetic pathway of the first-order phase transition such as crystalline ordering.

## RESULTS

### Experiments

**Fig. 1:**
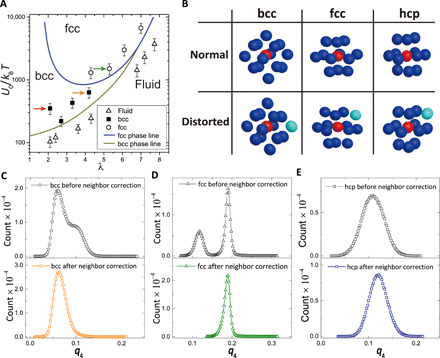
Examining local orderings under distortions caused by the neighbor particle fluctuation. (**A**) Phase diagram of our system as the function of λ and *U*_0_ (see Materials and Methods). The three arrows indicate the three system conditions discussed in the main text. (**B**) Illustration of experimentally observed regular and distorted local structures of bcc, fcc, and hexagonal close-packed (hcp) particles. Neighbor particle fluctuations distort the local structure through changing the neighbor number *Nb* within the cutoff distance *r_c_* of the first neighbor shell. Apparently, without destructing the structural similarity, the fluctuations move the blue particle out of (see bcc panel) or into (see fcc and hcp panels) the first neighbor shell of the red particle, resulting in ambiguity of local ordering identification with the bond-orientational order parameters. (**C** to **E**) Illustration of the effectiveness of the neighbor-correction procedure to identify bcc, fcc, and hcp local orders separately. The *q*_4_ distributions, which are wide and not unimodal due to the neighbor fluctuations before neighbor correction, become unimodal after neighbor correction, reflecting the well-defined structures.

Our experimental samples consist of poly(methyl methacrylate) PMMA colloidal particles with low polydispersity and density-matched and refractive index–matched polar solvents (see Materials and Methods). Those PMMA particles are negatively charged and interact with each other via weakly screened Coulomb repulsion. By changing the particle volume fraction, varying the relative ratio of solvent components, or vibrating the polar solvent with an ultrasonicator, we can obtain body-centered cubic (bcc)–stable, face-centered cubic (fcc)–stable, and fluid-stable samples. The tunability of the stable states offers the opportunity to study the kinetic processes of various types of transitions between them. Through conductivity measurements and electrophoretic measurements, we characterize our samples with the effective inverse screening length λ and the potential strength *U*_0_ (see Materials and Methods) and present the phase diagram on the λ − (*U*_0_/*k*_B_*T*) (*k*_B_*T*, thermal energy) plane in Fig. 1A, which is convenient for comparison with simulation works of hard-core Yukawa systems (*53*). We can see that the phase diagram has a typical polymorphic character. Thus, our system serves as a model for studying competing orderings in polymorphic systems. The effective temperature of the system is about 0.6 *T*_m_ ∼ 0.8 *T*_m_ (*T*_m_, the effective melting temperature) (see Materials and Methods). The details of image recording are described in Materials and Methods. We also use a hard sphere–like system to show the generality of our scenario (see Materials and Methods).

### Methods to reveal local structural orderings under thermal distortion

Here, we explain two important improvements of the original bond-orientational order approaches reported in our previous works to avoid misassignment of the local symmetry due to thermal structural distortion. One is based on a neighbor-correction method (*14*), which focuses on local structure information inside the first neighbor shell. The other is based on a Voronoi-weighted method (*17*, *54*), which takes into account information from a longer length scale.

We first explain our neighbor-correction method, which is introduced to remove the ambiguity due to neighbor-number fluctuations. In this method, on the basis of the assumption that structural distortions are due to thermal fluctuations and thus weak, we introduce a simple criterion to assign the correct neighbor number *Nb* of each particle, which leads to the correct identification of local structures. Many types of local orderings are formed in a supercooled liquid. Upon crystallization, they mutually compete and evolve into intermediate structures. Because of thermal positional fluctuations due to the lack of translational order and interpenetration of different polymorphs, the local orders inside intermediate structures are usually distorted from well-defined crystalline structures. The most common result of such distortion in our system is the formation of 13-bonded distorted structures, which correspond to none of the relevant crystalline orders in this system. Figure 1B illustrates the normal and distorted bcc, hexagonal close-packed (hcp), and fcc local structures of crystal nuclei in our experimental samples at λ = 4.1 (see the orange arrow in Fig. 1A). In these particular examples, the bcc panel shows that thermal fluctuations distort normal bcc [with 14 neighbors including 8 nearest ones and the other 6 second-nearest ones within the first minimum distance of the radial distribution function *g*(*r*)] through pushing the 14th nearest neighbor (light green sphere) of the central particle (red sphere) into the second neighbor shell. We can also see in Fig. 1B that thermal fluctuations also distort normal hcp and fcc [with 12 neighbors within the first minimum distance of the radial distribution function *g*(*r*)] through pulling the13th neighboring particles (light green spheres in hcp and fcc panels) into the first neighbor shell. Both types of distortions change different distorted crystalline local orders into 13-neighbor configurations, which produce ambiguous order parameters. If we use the conventional bond-orientational order approach, then they are incorrectly identified as amorphous configurations, although they are linked to crystalline orders. On noting that the relevant crystalline structures in our system have either 14 or 12 neighbors, we categorize these 13-neighbor configurations back into 14-neighbor or 12-neighbor ones. We do this by comparing the average of the distances from the central particle to its 14th neighbor (*r*_14_) and its 13th neighbor (*r*_13_), (*r*_13_ + *r*_14_)/2, with the first minimum distance of *g*(*r*), *r_c_*. The relevance of this choice of *r_c_* is shown in fig. S1. The underlying assumption rationalizing this criterion is that the distortion is just a consequence of thermal positional fluctuation of a stable configuration and thus has only perturbative (i.e., nondestructive) effects on local orderings. According to this criterion, the 14th neighbor particle (light green sphere) in the bcc panel of Fig. 1B is only slightly distorted and thus (*r*_13_ + *r*_14_)/2 < *r_c_* in this case. So, we assign the 14th neighbor particle to be the neighboring particle. Similarly, the 13th neighbor particle in hcp and fcc panels of Fig. 1B, for which (*r*_13_ + *r*_14_)/2 > *r_c_*, is assigned to be a non-neighboring particle. After the neighbor-correction procedure, locally distorted crystalline structures are successfully identified as correct ones by using the standard bond-orientational order parameters *q_l_* and *w_l_*. Here, *q_l_* and *w_l_* are rotationally invariant scalar quantities constructed from orientational relationship between particle and its neighbors, whose values present symmetry information of local clusters (see Materials and Methods for more details).

Figure 1 (C to E) demonstrates the effectiveness of this approach. In the upper panel of Fig. 1C, regular (14-neighbor) and distorted (13-neighbor) bcc configurations have separated *q*_4_ peaks before neighbor correction, which interferes with the bcc-type structure identification. Because regular and distorted bcc configurations still have large orientational similarity, their *q*_4_ values merge to a single bcc-characteristic peak after neighbor correction, as shown in the lower panel of Fig. 1C. Correspondingly, hcp and fcc configurations also have unimodal and characteristic *q*_4_ peaks after neighbor correction, as shown in the lower panels of Fig. 1 (D and E). These unimodal distributions corresponding to values of well-defined structures demonstrate the intrinsic structural similarity of the regular and distorted local orders under small distortions and the validity of our local structure identification method. By examining the bond-orientational order parameters *q_l_* and *w_l_* after the neighbor correction, each type of crystalline structures, irrespective of whether they are regular or distorted, presents a unimodal distribution characteristic to them, which allows unambiguous structure identifications even in a situation where many types of local orders coexist and compete under thermal fluctuations.

It should be noted that the above neighbor-correction procedure relies on rather small distortions of local orderings. In the range of 3.7 < λ < 7.5 and the effective system temperature 0.6 *T*_m_ < *T* < 0.8 *T*_m_, distortions of bcc-type, hcp-type, and fcc-type local orderings are induced mainly by competition between different types of order upon homogeneous crystal nucleation, and they are rather small. Thus, the neighbor-correction procedure is effective, allowing unambiguous structure identifications. However, at a low value of λ, for example, λ = 2.0 indicated by the red arrow in Fig. 1A, we find that distortions of the bcc-type local ordering are fairly large because of large interparticle distance fluctuations reflecting the very soft interparticle interaction. Accordingly, the neighbor-correction procedure does not work efficiently.

This indicates that, besides the neighbor-number fluctuation, we need to consider thermal distortions due to neighbor-length fluctuations. To do so, we use the Voronoi-weighted bond-orientational order approach (*17*, *54*), in which we consider the distortion of the whole Voronoi cell in calculating ${\overset{–}{q}}_{l}\prime$ and ${\overset{–}{w}}_{l}\prime$ (see Materials and Methods). This method uses a Voronoi cell construction to make the list of neighbors, including even particles sitting outside the first shell as neighbors. It does not treat the information on the distance of a neighbor particle to the central one in a direct manner but treat the spatial information of all neighboring particles by introducing a weight proportional to the area of each Voronoi face. Because of this selection method of neighbors, it is not so sensitive to the orientational characteristics of local structural orderings, particularly when different structures spatially coexist. To remove this weakness, we coarse-grained this Voronoi-weighted bond-orientational order parameters up to neighbors, to take the spatial extension of local orders into account. We find that the resulting coarse-grained Voronoi-weighted bond-orientational order parameters, ${\overset{–}{q}}_{l}\prime$ and ${\overset{–}{w}}_{l}\prime$, provide accurate structural information about the crystalline type, even when the system suffers from fairly large distance fluctuations.

With these improved bond-orientational order parameters, we can now discuss both crystalline (${\overset{–}{q}}_{6}\prime >0.35$) and relatively ordered precursor clusters ($0.27<{\overset{–}{q}}_{6}\prime <0.35$) formed in the early stage even for a system with large positional fluctuations. In the following, we look into local orderings of particles inside those clusters, taking advantage of the neighbor-corrected bond-orientational order parameters, *q_l_* and *w_l_*.

### Competing local structural orderings in the early stage of crystallization

**Fig. 2:**
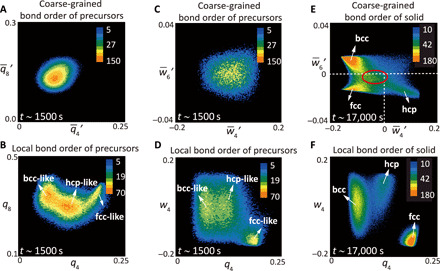
Coexisting bcc, hcp, and fcc local orders during crystallization. (**A**) ${\overset{–}{q}}_{4}\prime -{\overset{–}{q}}_{8}\prime$ distribution of relatively ordered precursor particles ($0.27<{\overset{–}{q}}_{6}\prime <0.35$) in the early stage [*t* ∼ 1500 s (278 τ_B_), φ_solid_ ∼ 5%], with only one patch. (**B**) *q*_4_ − *q*_8_ distribution of relatively ordered precursor particles ($0.27<{\overset{–}{q}}_{6}\prime <0.35$) in the early stage [the same as (A)]. We can see three patches corresponding to different local orders. (**C**) ${\overset{–}{w}}_{4}^{\prime }-{\overset{–}{w}}_{6}^{\prime }$ distribution of relatively ordered precursor particles ($0.27<{\overset{–}{q}}_{6}\prime <0.35$) in the early stage [the same as (A) and (B)], with only one patch. (**D**) *q*_4_ − *w*_4_ distribution of relatively ordered precursor particles ($0.27<{\overset{–}{q}}_{6}\prime <0.35$) in the early stage [the same as (A) to (C)]. We can see three patches corresponding to different local orders. (**E**) ${\overset{–}{w}}_{4}^{\prime }-{\overset{–}{w}}_{6}^{\prime }$ distribution of crystalline particles ($0.35<{\overset{–}{q}}_{6}\prime$) in the late stage [*t* ∼ 17,000 s (3148 τ_B_), φ_solid_ ∼ 90%]. We can see three cores well corresponding to bcc, fcc, and hcp. However, the three patches overlap and interweave around ${\overset{–}{w}}_{4}^{\prime }=0$ and ${\overset{–}{w}}_{6}^{\prime }=0$ (circumscribed by red circle), indicating the existence of intermediate structures even at late stage. (**F**) *q*_4_ − *w*_4_ distribution of crystalline particles ($0.35<{\overset{–}{q}}_{6}\prime$) at the late stage [the same as (E)]. We can see three patches corresponding to bcc, fcc, and hcp local orders.

**Fig. 3:**
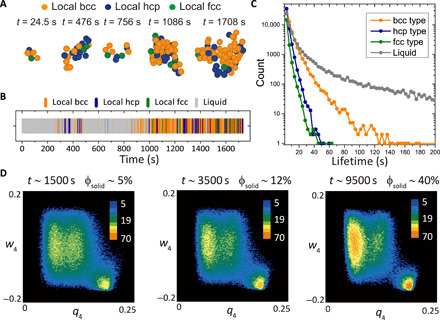
Fluctuation of local orders at the early stage of crystal nucleation. (**A**) Real-space time evolution of local orders within a typical relatively ordered cluster, containing precursors and crystal nuclei during the nucleation process at λ = 4.1, where bcc is stable (see the orange arrow in Fig. 1A). The local order is spatiotemporally fluctuating among bcc, hcp, and fcc types. (**B**) Temporal fluctuation of local order of a typical particle in the cluster. The local order is frequently fluctuating among bcc, hcp, and fcc types. (**C**) Residence time distribution of each type of local order in a supercooled liquid in the nucleation stage. We can see a long residence time of bcc-like local order, which promotes the nucleation of bcc crystals. (**D**) *q*_4_ − *w*_4_ distribution of relatively ordered particles ($0.27<{\overset{–}{q}}_{6}\prime$) at different time during the early stage of crystallization.

By using the above-described analysis methods, we first focus on local orderings in the nucleation and early growth stages. Precursors with high orientational order are important intermediates during crystal nucleation. With the bond-orientational order parameters *q_l_*, *w_l_*, and ${\overset{–}{w}}_{l}\prime$, after removing thermal distortions, we reveal the coexistence of competing local orders during crystal nucleation (Fig. 2, A to D) and observe that crystal nuclei emerge from precursor clusters, containing different types of local orderings with temporal fluctuations (Fig. 3). As pointed out by previous simulations and experiments, a precursor cluster is ordered only partially, and its relatively well-ordered parts trigger nucleation of crystals (*55*, *56*). In our experiments of a sample indicated by orange arrow in Fig. 1A, we pick up precursor particles ($0.27<{\overset{–}{q}}_{6}\prime <0.35$) in the nucleation stage (around *t* ∼ 1500 s ∼ 278 τ_B_ after shear melt) at φ_solid_ ∼ 5% and carefully characterize their local structures. Here, τ_B_ ∼ 5.4 s is the Brownian time of a colloid particle, $\frac{\eta \sigma^{3}}{k_{B}T}$, where η is the solvent viscosity and σ is the particle diameter. As shown in Fig. 2 (A and C), both ${\overset{–}{w}}_{4}\prime -{\overset{–}{w}}_{6}\prime$ and ${\overset{–}{q}}_{4}\prime -{\overset{–}{q}}_{8}\prime$ maps of these particles ($0.27<{\overset{–}{q}}_{6}\prime <0.35$) have only one featureless patch, indicating the liquid-like disordered nature of precursor clusters in the medium-range (second neighbor) length scale. However, once looking at the nearest neighbor arrangement by our new method, we are able to see the presence of three distinct patches in the *q*_4_ − *w*_4_ and *q*_4_ − *q*_8_ maps of these particles ($0.27<{\overset{–}{q}}_{6}\prime <0.35$), as shown in Fig. 2 (B and D), indicating that the precursor clusters actually consist of three types of local orders. These local orders are consistent with the crystalline orders of bcc, fcc, and hcp types. However, we find that the local orders are spatially mixed inside the precursor clusters, which precludes forming spatially extended positional order [see the comparison of the pair correlation function *g*(*r*) between precursors and crystals in fig. S2]. Moreover, considering that the ordered particles belonging to precursors are also accompanied by other liquid-like particles (${\overset{–}{q}}_{6}\prime <0.27$), the complex disordered nature of precursors is generated by coexistence of various types of local orders, in which various orders are spatially mixed and temporally fluctuating. This is consistent with a picture that precursors are not crystals and a consequence of local structural fluctuations intrinsic to a supercooled liquid (*36*).

Here, it may be worth mentioning the characteristic feature of precursors. We find that the cluster size probability distribution *P*(*N*) obeys a power-law decay *P*(*N*) ∝ *N* ^−1.7^, with the cluster size *N* (see fig. S3). We note that precursors of *N* ≳ 40 include solid particles in their inside. Thus, the genuine precursors have a size less than about four-particle diameter. The power-law decay of the precursor size distribution implies the stochastic nature of precursor formation.

We also found strong temporal fluctuations of these local orders during the entire nucleation process. Figure 3A illustrates the real-space time evolution of local orderings within a typical relatively ordered cluster ($0.27<{\overset{–}{q}}_{6}\prime$) containing precursors and crystal nuclei, which are observed in a bcc-stable sample at λ = 4.1 during the nucleation process. Once the lifetime of ordered clusters (${\overset{–}{q}}_{6}\prime >0.35$) becomes long enough, we are able to directly calculate its probability of shrink and growth experimentally. When the two probabilities become comparable, the ordered clusters are identified to reach about the critical nucleus size. Until a bcc-type nucleus reaches the critical nucleus size at *t* = 1708 s (316 τ_B_), the three types of crystalline local orders coexist and frequently fluctuate among them. Temporal fluctuation of the local order of a typical particle in the cluster is illustrated in Fig. 3B. Except for the prevailing bcc-type order, we observe that the particle experiences many short–time scale fluctuations among the three types of local orders. This feature is also seen in all particles’ residence time distribution for each type of local order in the relative ordered clusters before reaching the critical nucleus size, as shown in Fig. 3C. The longer residence time of bcc-type local order, which is a manifestation of the fact that the bcc-type order is most stable, indicates the better spatial coherency, which promotes the formation of a bcc-type critical nucleus. On the other hand, the short–time scale fluctuation is a manifestation of the liquid-like unstable nature of the precursor clusters.

To see the generality of our scenario, we also perform crystallization experiments in a hard sphere–like system (volume fraction, φ ∼ 54%) (see Materials and Methods for the system and fig. S4 for the results). We observe a similar nucleation behavior: The precursor stage is characterized by the spatially mixed orders and temporal fluctuations among them. Then, in the later stage, the lifetime and the spatial coherency of fcc-type order gradually increase with time, promoting the nucleation of the most stable fcc solids (see fig. S4).

The temporal increase in the stability of bcc-type order in charged colloidal systems and fcc-type order in hard sphere–like systems, which we may regard as the elementary process of symmetry selection in the nucleation process, can be seen as cross-symmetry transformations observed in previous work (*14*). The only difference between charged colloidal systems and hard sphere–like systems is the difference in the most stable solid polymorph (bcc versus fcc, respectively), which leads to the difference in the cross-symmetry transformation path (to bcc versus to fcc, respectively). This suggests the generality of our scenario, although further study is necessary to firmly establish it.

We also look at the growth front of a postcritical nucleus. Particles on the growth front exhibit nearly the same characteristics as precursors in Fig. 3 (B and C): the spatial coexistence and strong temporal fluctuation of different local orders. These features are generally seen in the early stage of crystallization until particles are deeply wrapped inside crystalline clusters (i.e., involved in well-developed translational order). Figure 3D demonstrates the behavior of relatively ordered particles ($0.27<{\overset{–}{q}}_{6}\prime$) at different times during the early stage of crystallization. As more and more particles are wrapped into crystalline clusters, the fluctuation among different local orders becomes less frequent. This leads to the transition from the liquid-like dynamical state to a solid-like stable one.

### Competing local structural orderings in the late growth stage

**Fig. 4:**
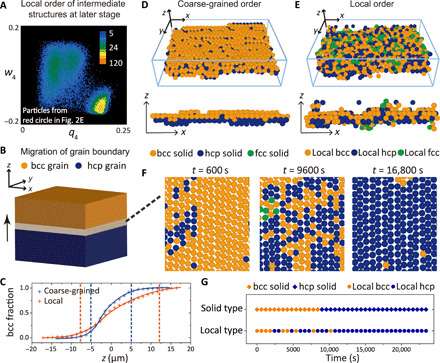
Local orderings in the solid-solid boundaries. (**A**) *q*_4_ − *w*_4_ distribution of particles at the interweaving zone in Fig. 2E. Three divisional patches corresponding to bcc-, fcc-, and hcp-type local orders are observed. (**B**) Schematic representation of the migration of a typical hcp-bcc boundary. (**C**) Change of the bcc fraction along *z* direction across the interface, showing a thick asymmetric boundary. (**D**) Real-space 3D representation of hcp-bcc interface seen by the coarse-grained order parameter.We can see a sharp two-layer interface. (**E**) Real-space 3D representation of the same hcp-bcc interface as in (D), seen by the local order parameter. Unlike in (D), we can see a diffuse thick interface where different local orders coexist. (**F**) Time evolution of local orders inside a particle layer parallel to the boundary [*x* − *y* plane; see (B)]. (**G**) Temporal fluctuation of coarse-grained and local orders of a typical particle.

Now, we turn our attention to the boundary migration behavior at the late stage of crystallization. At this stage, solid particles make up more than 85% and exist in forms of comparatively large grains of different symmetries, but the most stable bcc grains are gradually growing through grain-boundary migration. In this stage, abundant solid-solid boundaries are present, which can be regarded as another type of intermediate structures. As shown in Fig. 2E, the ${\overset{–}{w}}_{4}^{\prime }-{\overset{–}{w}}_{6}^{\prime }$ distribution of solid particles ($0.35<{\overset{–}{q}}_{6}\prime$) in the late stage (*t* ∼ 17,000 s, φ_solid_ ∼ 90%) already shows three distinct cores, corresponding to the three crystalline types. However, the three patches on this coarse-grained order parameter map still spread and have a notable overlap region, forming an intermediate zone with a considerable number of particles around ${\overset{–}{w}}_{4}^{\prime }=0$ and ${\overset{–}{w}}_{6}^{\prime }=0$ (red circled region). Here, it is worth pointing out that the *q*_4_ − *w*_4_ map of local orderings in Fig. 2F presents more separated patches. Particles in the intermediate zone in these maps are regarded as intermediate structures in the late stage and mainly composed of particles in the boundaries. In Fig. 4A, we present particles from this overlap region of the *q*_4_ − *w*_4_ map, together with the information on their local orders. Particles from the featureless interweaving region are actually composed of three separated patches corresponding to bcc, fcc, and hcp local orderings, indicating that solid-solid boundaries formed in the late stage consist of competing local orders.

To probe the structural characteristics of boundary intermediates, we investigate the migration of a typical hcp-bcc interface (parallel to *x*-*y* plane) in an fcc-stable sample at λ = 5.4 (see the green arrow in Fig. 1A). The result is shown in Fig. 4B. We separately categorize the particle type with coarse-grained and local order parameters (see Materials and Methods). We also show in Fig. 4C the change of the bcc fraction along *z* direction across the interface. If we define the region between 90% fraction to 10% fraction as the interface (see the vertical dashed lines), then the local order has a thicker and more asymmetric interface profile compared to the coarse-grained one. To see this feature in more detail, we show the three-dimensional (3D) distribution of the boundary particles in Fig. 4 (D and E). The boundary particle is defined as a particle more than 30% of whose neighbors have different types of symmetry: For example, a bcc particle has 14 neighbors, and thus, if more than 5 neighbors are not bcc type, then this particle is assigned as a boundary particle. The coarse-grained order exhibits a sharp and clear two-layer boundary (see Fig. 4D), whereas the local order exhibits a boundary with rough and diffuse morphology due to competing local orders (see Fig. 4E). This indicates that the local order strongly fluctuates in the solid-solid interface even in the late stage. The small structural coherency across the boundaries is responsible for the diffuse interface, leading to a low interface tension.

The above finding strongly suggests that the fluctuation of local orders inside the diffuse boundary plays a key role in the boundary migration and thus, the structural evolution toward the stable crystalline phase. This is confirmed by the following dynamical analysis of the migration of the boundary (see Fig. 4B), which takes place along *z* direction (see the arrow). Figure 4F illustrates the temporal change of the local orders in a single-particle layer at a fixed *z* position (see the gray-color layer in Fig. 4B) during the boundary migration process. Strong spatiotemporal fluctuation of local orders is observed in the layer when the grain boundary passes through it. The boundary reaches the particle layer around *t* = 1000 s (185 τ_B_) and leaves from it around *t* = 14,000 s (2593 τ_B_). The local order fluctuations are notably reduced around *t* = 16,800 s (3111 τ_B_) when the solid-solid boundary leaves far away from that layer. Temporal fluctuations of the coarse-grained and local orders of a typical particle are also illustrated in Fig. 4G. We can see that, before the particle stably converts to an hcp-type solid structure, its local order switches several times and eventually triggers the migration event. Microscopically, the boundary migration is realized by a heterogeneous slippery motion of layered particles (see Fig. 4F), resulting in the fluctuations of local orders and thus leading to a diffuse interface.

After the long-time competition among local orders, the system evolves to a polycrystalline state, where rhcp (a mixture of fcc and hcp) polymorph is dominant. A large-field observation tells us that most of the grain boundaries are not flat at any time. The capillary wave of the large-angle grain boundaries (LAGB) in our system has a correlation length of about 30 interparticle distances. These results suggest that our system is near or above the roughening temperature of LAGBs, which may allow our grain-boundary migration modes to be active.

## CONCLUSION

To summarize, we study how polymorph selection and the formation of the final polycrystalline structure are made under competing orderings in systems with polymorphism. This problem is of critical importance not only for our fundamental understanding of crystallization phenomena but also for the application to polymorph control of functional materials. Colloidal systems allow us to experimentally access both particle-level structures and dynamics. This accessibility to the elemental ordering process and the improvement of microscopic structural analysis based on bond-orientational order parameters enable us to efficiently identify local orders even under thermally excited positional fluctuations and thus, to uncover physically relevant local structural information of the intermediate states of crystallization such as precursors, metastable solids, and grain boundaries.

At a macroscopic level, a liquid-to-crystal transition is a discontinuous first-order transition. By removing thermal positional fluctuations by our new method, we are able to reveal bond-orientational orders linked to underlying polymorphs and access spatiotemporal fluctuations among them at a particle level. This allows us to address how crystalline ordering takes place under the competition among different polymorphs at a microscopic level. The birth of crystal nuclei from a liquid starts from crystalline precursors, which are regions of high crystalline bond-orientational orders spontaneously formed in a supercooled liquid. Initially, local orders linked to various polymorphs coexist and fluctuate among them in precursors. Then, the most stable polymorph gradually becomes more and more dominant while accompanying the increase in its spatial coherence and lifetime. We also find that the late stage of crystallization, which is driven by migration of grain boundaries, also has a similar feature: Transformation of a less stable polymorph to a more stable one takes place “not” in a direct manner but through a mixed state of competing orders, which form grain boundaries.

Our study shows that both the symmetry selection in the early stage of crystallization and the growth of domains of the most stable crystal in the late stage are not deterministic processes but rather continuous stochastic processes toward the most stable polymorph under competition with the others. This indicates that crystalline ordering is intrinsically stochastic at a microscopic level: It is characterized by spatiotemporal fluctuations among polymorph-related orientational symmetries and the gradual increase of the lifetime and spatial coherency of the most stable one, reflecting the stability difference among different polymorphs. Only when the spatial coherency becomes long enough to sustain translational order over a long distance that order-parameter fluctuations are suppressed.

Our study experimentally establishes a particle-level picture of the elementary kinetic pathway of colloidal crystallization. Thus, it sheds new light on the crystallization kinetics from a microscopic perspective. We expect that the basic physical scenario we found here may be generic to crystallization in a wide class of systems with polymorphism.

## MATERIALS AND METHODS

### Sample preparation

#### Charged colloidal systems

We suspend DiIC_18_ (Dioctadecyl-3,3,3′,3′-tetramethylindocarbocyanine perchlorate)–dyed PMMA colloids grafted with polyhydroxystearic acid polymer in a mixture of the nonpolar solvent (tetrahydronaphthalene, Sigma-Aldrich) and the weakly polar solvent (mixture of iododecane and iodododecane, Sigma-Aldrich), whose refractive index and density are closely matched with those of particles, inside a glass capillary. The PMMA particles, which have diameter σ = 2.2 μm with the polydispersity of less than 2.5%, are negatively charged in the solvent and interact with each other via a weakly screened Coulomb repulsion. In this study, the colloidal particle volume fraction ϕ is limited below 35%, where particles do not experience direct contacts and can be safely assumed to interact with the soft repulsive interaction.

To compare with the previously reported hard-sphere Yukawa phase diagram (*53*), we use the variables of λ and *U*_0_, which are calculated from the Debye screening length κ and the surface charge *Z* as λ = κσ/(6ϕ/π)^−1/3^ and *U*_0_ = *e*^κσ^ε(6ϕ/π)^1/3^. Here, ε = *Z*^2^*e*^2^/4πε_s_[σ(1 + κσ/2)^2^], with ε_s_ being the dielectric constant of the solvent. The screening length κ^−1^ is acquired from the conductivity measurements (*14*, *57*–*59*), and the surface charge *Z* is estimated from the electrophoretic measurements (*57*, *60*–*62*). By varying the relative fractions of the two components of the solvent, we can adjust the Debye screening length κ^−1^ and thus tune the stable solid state. The phase diagram is established from the measurements of various samples, as shown in Fig. 1A.

According to the distance to the melting line in the phase diagram, the effective temperature of the system are estimated to be around 0.6 *T*_m_ ∼ 0.8 *T*_m_, where *T*_m_ is the effective melting temperature in our system. We also calculate the Lindemann parameter [$\Delta L={\langle {\left(r_{i}(t)-r_{i}\right)}^{2}\rangle }^{1/2}/d$, where *d* is the average neighbor distance] of a crystal before shear melting. Our particle-tracking measurements yield Δ*L* ∼ 0.105, which is consistent with the effective system temperature estimated from the phase diagram (*63*).

We shear-melt colloidal crystals with a magnetic wire to form a homogeneous liquid state and then follow the crystallization process with a Leica SP8 fast confocal microscope immediately after the agitation. The scanning speeds (∼10 μm/s along *z* direction, with a 150 μm by 150 μm *x*-*y* view field) in all dimensions are at least 24 times faster than the Brownian time (∼5.4 s), i.e., the time for a particle to freely diffuse over the particle size (2.2 μm), which enables the precise positioning and dynamical tracking of particles. For a good statistics like in Fig. 2, we use a typical imaging stack having the *x* × *y* × *z* dimensions of 150 μm by 150 μm by 100 μm and containing about 100,000 particles. For a higher time resolution (Δ*t* ∼ 2 s), on the other hand, we typically set the imaging stack dimension as 150 μm by 75 μm by 50 μm.

### Hard sphere–like systems

We suspend Nile Red–dyed PMMA colloids and polyhydroxystearic acid polymer–grafted PMMA colloids in a mixture of *cis*-decahydronaphthalene and tetrachloroethylene (Sigma-Aldrich), whose refractive index and density are closely matched with those of particles, inside a glass capillary. The PMMA particles have diameter σ = 1.8 μm, with the polydispersity of less than 2.5%. The colloidal particle volume fraction ϕ is around 54%. We find that adding 50 mM/liter dioctyl sulfosuccinate sodium salt (Sigma-Aldrich) to the system largely screens surface charges on particles, which allows us to regard the system as hard-sphere like. The other experimental procedures are the same as the charged colloidal systems.

### Structural parameters for proper identification of local orders

First, we eliminate the 13-bond particles generated by neighbor-number fluctuation by the method described in the main text and make the modified neighbor list *Nb*(*i*). Then, we calculate $q_{l}(i)={\left(\frac{4\pi }{2l+1}\sum_{m=-l}^{l}{|q_{l,m}(i)|}^{2}\right)}^{1/2}$, and $w_{l}(i)=\sum_{m_{1}+m_{2}+m_{3}=0}^{l}(\begin{array}{ccc} l & l & l\\ m_{1} & m_{2} & m_{3} \end{array})\frac{q_{l,m_{1}}(i)q_{l,m_{2}}(i)q_{l,m_{3}}(i)}{{|q_{l}(i)|}^{3}}$, with $q_{l,m}(i)=\frac{1}{\mathrm{Nb}(i)}\sum_{j=1}^{\mathrm{Nb}(i)}Y_{l,m}(\theta_{i,j},\phi_{i,j})$. Here, *Y*_*l*,*m*_(θ_*i*,*j*_, ϕ_*i*,*j*_) are spherical harmonics functions with ϕ *m* ∈ [−*l*, *l*], where θ_*i*,*j*_ and ϕ_*i*,*j*_ are the polar and azimuthal angles of the vector **r**_*i*,*j*_ pointing from the center particle *i* to any particle *j* of its *Nb*(*i*) neighbors, respectively, and the parenthesis term is the Wigner-3j symbol. Then, the local order identification is made as follows: If the order parameters of a particle are located on the left side of line between (0.125, −0.2) and (0.25, 0.2) in the *q*_4_ − *w*_4_ map, then the particle is identified as local bcc for *Nb* = 14 and local hcp for *Nb* = 12. Otherwise, it is identified as local fcc for *Nb* = 12.

### Structural parameters for identification of coarse-grained orders

We first characterize the structural order of a particle by parameters ${\overset{–}{q}}_{l,m}^{\prime }(i)=\sum_{f\in F(i)}\frac{A(f)}{A}Y_{l,m}(\theta_{i,j},\phi_{i,j})$, where *A*(*f*) is the surface area of the Voronoi cell facet *f* separating center particle *i* and its neighbor *j*, and *A* is the total surface area of the Voronoi cell boundary *F*(*i*). So, the neighbor list is determined by Voronoi construction, instead of a distance criterion. Then, the coarse-grained structural parameters, ${\overset{–}{q}}_{l}\prime$ and ${\overset{–}{w}}_{l}\prime$, are obtained as ${\overset{–}{q}}_{l,m}^{\prime }=\frac{1}{\mathrm{Nb}\prime }\Sigma_{k=0}^{\mathrm{Nb}\prime }{\overset{–}{q}}_{l,m}^{\prime }(k)$, ${\overset{–}{q}}_{l}^{\prime }(i)={\left(\frac{4\pi }{2l+1}\sum_{m=-l}^{l}{|{\overset{–}{q}}_{l,m}^{\prime }(i)|}^{2}\right)}^{1/2}$ and ${\overset{–}{w}}_{l}^{\prime }(i)=\sum_{m_{1}+m_{2}+m_{3}=0}^{l}(\begin{array}{ccc} l & l & l\\ m_{1} & m_{2} & m_{3} \end{array})\frac{{\overset{–}{q}}_{l,m_{1}}^{\prime }(i){\overset{–}{q}}_{l,m_{2}}^{\prime }(i){\overset{–}{q}}_{l,m_{3}}^{\prime }(i)}{{\left(\sum_{m=-l}^{l}{|{\overset{–}{q}}_{l,m}^{\prime }(i)|}^{2}\right)}^{3/2}}$, where *Nb*^′^ is the neighbor number determined by Voronoi cell analysis. Then, the particle is identified as bcc solid if ${\overset{–}{w}}_{6}\prime >0$. ${\overset{–}{w}}_{4}\prime =0$ is not appropriate to separate fcc from hcp solid. Thus, we select particles with ${\overset{–}{w}}_{6}\prime <0$ and use the same line in the *q*_4_ − *w*_4_ map, which we used above, to separate between fcc and hcp.

## Supplementary Material

aaw8938_SM.pdf

## SUPPLEMENTARY MATERIALS

Supplementary material for this article is available at http://advances.sciencemag.org/cgi/content/full/6/27/eaaw8938/DC1
