# Supplementary material for: Revealing roles of competing local structural orderings in crystallization of polymorphic systems
Source: Sci Adv. 2020 Jul 1;6(27):eaaw8938. doi: 10.1126/sciadv.aaw8938 (PMC7329355; doi:10.1126/sciadv.aaw8938)
Supplement: aaw8938_SM.pdf [file aaw8938_SM.pdf]

Science Advances
AAAS

advances.sciencemag.org/cgi/content/full/6/27/eaaw8938/DC1

Supplementary Materials for

Revealing roles of competing local structural orderings in crystallization of polymorphic systems

Minhuan Li, Yanshuang Chen, Hajime Tanaka*, Peng Tan*

*Corresponding author. Email: tanaka@iis.u-tokyo.ac.jp (H.T.); tanpeng@fudan.edu.cn (P.T.)

Published 1 July 2020, *Sci. Adv.* **6**, eaaw8938 (2020)
DOI: 10.1126/sciadv.aaw8938

This PDF file includes:

Figs. S1 to S4

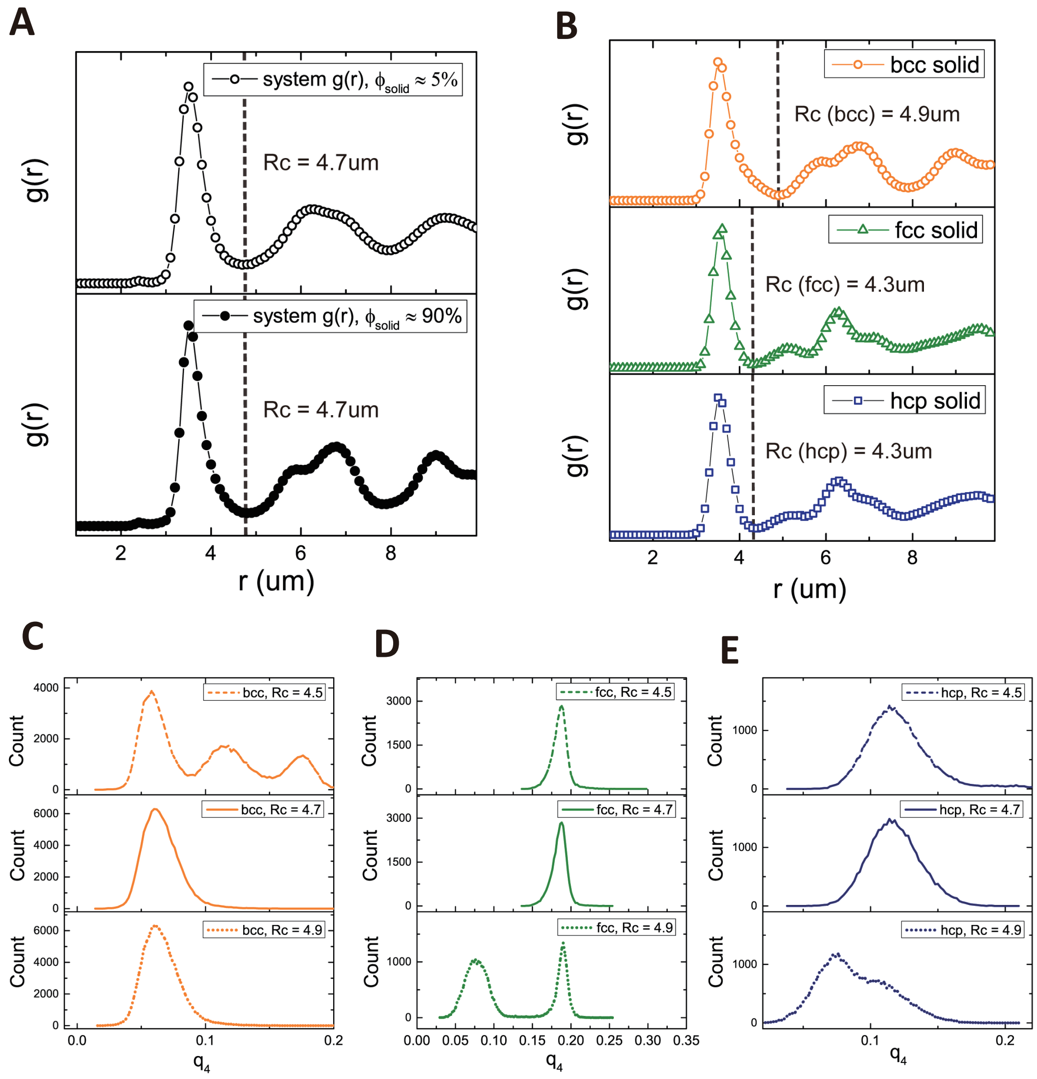

Figure S1: **The r_c-dependence of the bond split method.** (**A**) The radial distribution function $g(r)$ of a typical system with competing local orders in the nucleation (c) and later growth stage ($\phi_{\text{solid}} = 90$ %). The dashed line indicate the first minimum distance of $g(r)$. (**B**) The radial distribution function $g(r)$ of bcc, fcc and hcp solids (from top to bottom). The bcc solids have larger first minimum distance ($r = 4.9$ μm) of $g(r)$ than those of fcc and hcp solids ($r = 4.3$ μm). (**C**)-(**E**) Comparison between results with the choices of $r_c = 4.5$ μm, 4.7 μm and 4.9 μm respectively. A part of the bcc solids are mis-assigned as hcp and fcc solids with r_c=4.5 μm (shown in the top panel of **D**), whereas parts of the fcc and hcp solids are misassigned as bcc solids with r_c=4.9 μm (shown in the bottom panel of **D** and **E**). The choice of r_c=4.7 μm produces the sharpest q_4 distribution (i.e., with the narrowest half-width).

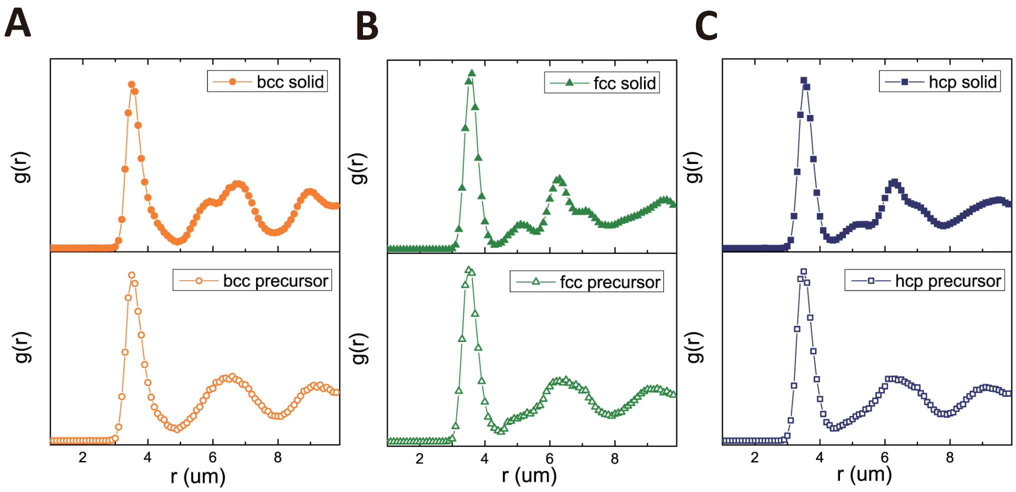

Figure S2: **Structural comparison between precursors and nuclei in the nucleation stage.** (**A**) Comparison of the radial distribution functions between bcc precursors and bcc solids. They have the similar first and second minimum distances of $g(r)$, but bcc precursors do not have the characteristic double peak structure in the second neighbor shell, unlike the bcc nuclei. (**B**) and (**C**) Similar comparisons for fcc and hcp structures respectively. These results all clearly indicate that the precursors are lack of translational order.

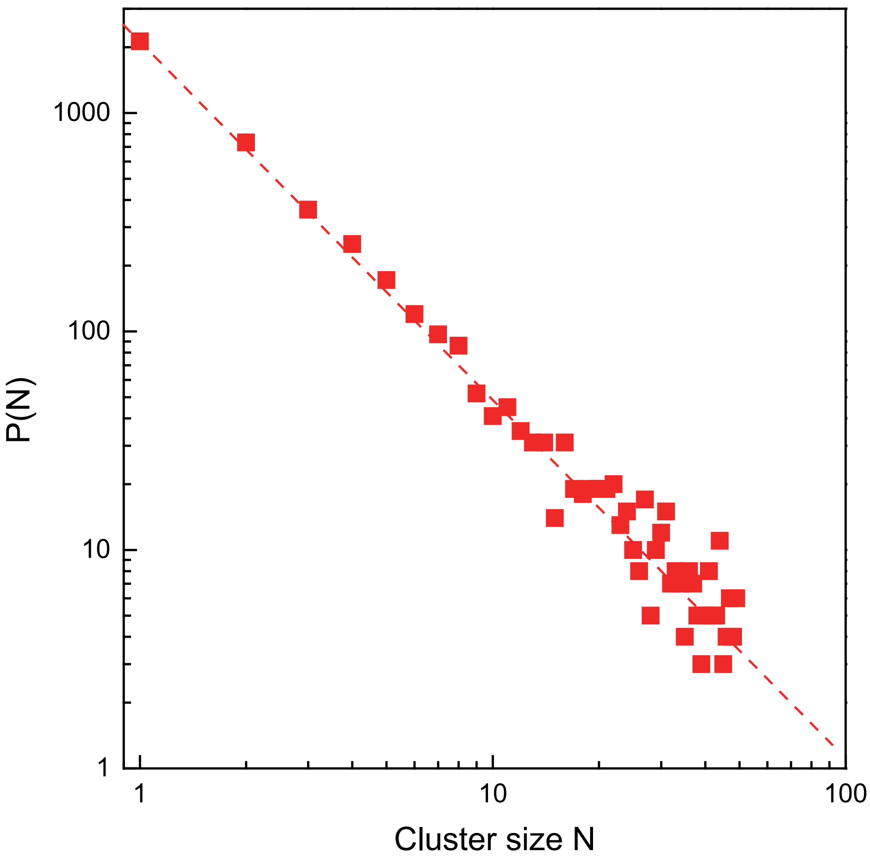

Figure S3: **Cluster size probability distribution of precursors in the very initial stage of nucleation ($t < 600$ s ($111\tau_B$), $\phi_{\text{solid}} < 1$ %).** The cluster size probability $P(N)$ has a distinct power law decay as a function of the cluster size N: $P(N) \propto N^{-1.7}$. It is worth mentioning that the largest genuine precursor cluster contains about 40 particles. Note that a precursor exceeding this size contains solid crystalline particles in its inside.

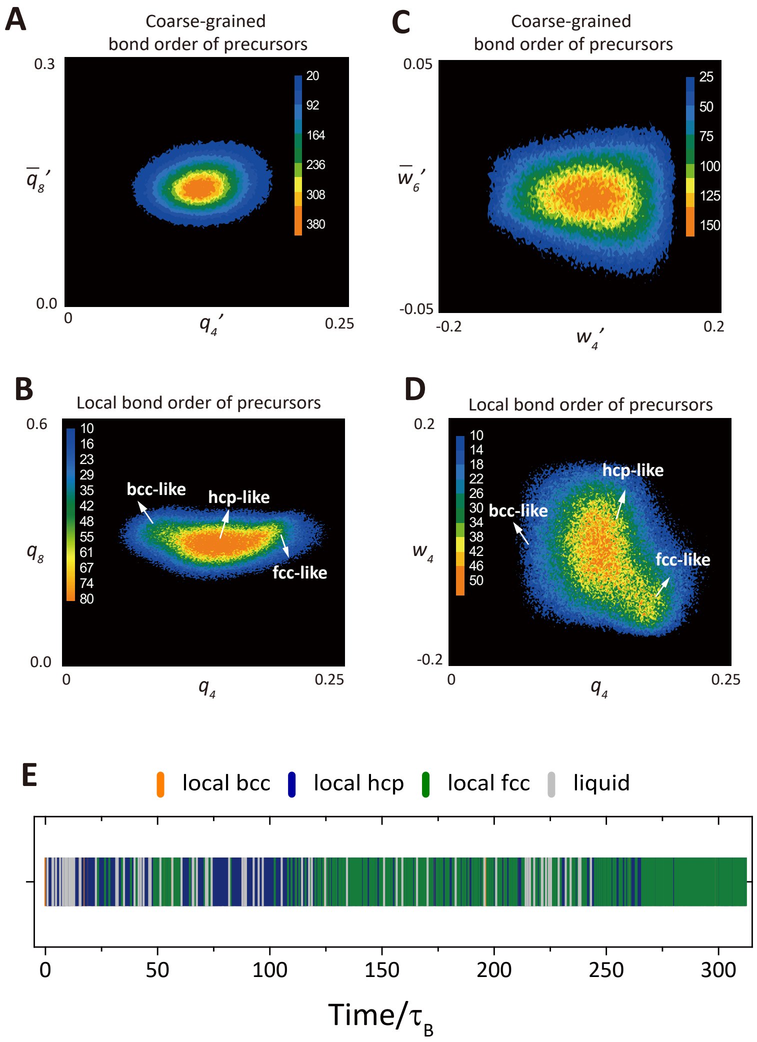

Figure S4: **Competing local orders and their temporal fluctuations in a hard-sphere-like system (volume fraction $\phi \sim 54$ %).** (**A**) The $\bar{q}'_4$-$\bar{q}'_8$ distribution of relatively ordered precursor particles ($0.27 < \bar{q}'_6 < 0.35$) in the early stage ($t \sim 300\tau_B$, $\phi_{\rm solid} \sim 3$ %), with only one patch. (**B**) The $q_4 - q_8$ distribution of relatively ordered precursor particles ($0.27 < \bar{q}'_6 < 0.35$) in the early stage. We can see two major patches corresponding to hcp-type and fcc-type local orders and very small bcc-like patch. (**C**) The $\bar{w}'_4 - \bar{w}'_6$ distribution of relatively ordered precursor particles ($0.27 < \bar{q}'_6 < 0.35$), with only one patch. (**D**) The $q_4 - w_4$ distribution of relatively ordered precursor particles ($0.27 < \bar{q}'_6 < 0.35$) in the early stage. We can see two major patches corresponding to hcp-type and fcc-type local orders and very small bcc-like patch. (**E**) Temporal fluctuations of local order of a typical particle during the nucleation stage. The local order is frequently fluctuating between hcp-type and fcc-type. A fcc-type nucleus is firstly observed around $t = 200\tau_B$. We can see that the long lifetime of fcc-type local order promotes its nucleation.
